# Associations on the Fly, a new feature aiming to facilitate exploration of the Open Targets Platform evidence

**DOI:** 10.1093/bioinformatics/btaf070

**Published:** 2025-02-12

**Authors:** Carlos Cruz-Castillo, Luca Fumis, Chintan Mehta, Ricardo Esteban Martinez-Osorio, Juan Maria Roldan-Romero, Helena Cornu, Prashant Uniyal, Antonio Solano-Roman, Miguel Carmona, David Ochoa, Ellen Mary McDonagh, Annalisa Buniello

**Affiliations:** European Molecular Biology Laboratory, European Bioinformatics Institute (EMBL-EBI), Wellcome Genome Campus, Hinxton, Cambridgeshire CB10 1SD, United Kingdom; Open Targets, Wellcome Genome Campus, Hinxton, Cambridgeshire CB10 1SD, United Kingdom; European Molecular Biology Laboratory, European Bioinformatics Institute (EMBL-EBI), Wellcome Genome Campus, Hinxton, Cambridgeshire CB10 1SD, United Kingdom; Open Targets, Wellcome Genome Campus, Hinxton, Cambridgeshire CB10 1SD, United Kingdom; European Molecular Biology Laboratory, European Bioinformatics Institute (EMBL-EBI), Wellcome Genome Campus, Hinxton, Cambridgeshire CB10 1SD, United Kingdom; Open Targets, Wellcome Genome Campus, Hinxton, Cambridgeshire CB10 1SD, United Kingdom; European Molecular Biology Laboratory, European Bioinformatics Institute (EMBL-EBI), Wellcome Genome Campus, Hinxton, Cambridgeshire CB10 1SD, United Kingdom; Open Targets, Wellcome Genome Campus, Hinxton, Cambridgeshire CB10 1SD, United Kingdom; European Molecular Biology Laboratory, European Bioinformatics Institute (EMBL-EBI), Wellcome Genome Campus, Hinxton, Cambridgeshire CB10 1SD, United Kingdom; Open Targets, Wellcome Genome Campus, Hinxton, Cambridgeshire CB10 1SD, United Kingdom; European Molecular Biology Laboratory, European Bioinformatics Institute (EMBL-EBI), Wellcome Genome Campus, Hinxton, Cambridgeshire CB10 1SD, United Kingdom; Open Targets, Wellcome Genome Campus, Hinxton, Cambridgeshire CB10 1SD, United Kingdom; European Molecular Biology Laboratory, European Bioinformatics Institute (EMBL-EBI), Wellcome Genome Campus, Hinxton, Cambridgeshire CB10 1SD, United Kingdom; Open Targets, Wellcome Genome Campus, Hinxton, Cambridgeshire CB10 1SD, United Kingdom; College of Arts, Media and Design, Northeastern University, Boston, MA, 02115, United States; Flatiron Health, New York, NY, 10010, United States; AstraZeneca UK Limited, Cambridge, CB2 0AA, United Kingdom; European Molecular Biology Laboratory, European Bioinformatics Institute (EMBL-EBI), Wellcome Genome Campus, Hinxton, Cambridgeshire CB10 1SD, United Kingdom; Open Targets, Wellcome Genome Campus, Hinxton, Cambridgeshire CB10 1SD, United Kingdom; European Molecular Biology Laboratory, European Bioinformatics Institute (EMBL-EBI), Wellcome Genome Campus, Hinxton, Cambridgeshire CB10 1SD, United Kingdom; Open Targets, Wellcome Genome Campus, Hinxton, Cambridgeshire CB10 1SD, United Kingdom; Wellcome Sanger Institute, Wellcome Genome Campus, Hinxton, Cambridgeshire, CB10 1SD, United Kingdom; European Molecular Biology Laboratory, European Bioinformatics Institute (EMBL-EBI), Wellcome Genome Campus, Hinxton, Cambridgeshire CB10 1SD, United Kingdom; Open Targets, Wellcome Genome Campus, Hinxton, Cambridgeshire CB10 1SD, United Kingdom

## Abstract

**Motivation:**

The Open Targets Platform (https://platform.opentargets.org) is a unique, comprehensive, open-source resource supporting systematic identification and prioritisation of targets for drug discovery. The Platform combines, harmonizes and integrates data from >20 diverse sources to provide target–disease associations, covering evidence derived from genetic associations, somatic mutations, known drugs, differential expression, animal models, pathways and systems biology. An in-house target identification scoring framework weighs the evidence from each data source and type, contributing to an overall score for each of the 7.8M target–disease associations. However, the old infrastructure did not allow user-led dynamic adjustments in the contribution of different evidence types for target prioritisation, a limitation frequently raised by our user community. Furthermore, the previous Platform user interface did not support navigation and exploration of the underlying target–disease evidence on the same page, occasionally making the user journey counterintuitive.

**Results:**

Here, we describe ‘Associations on the Fly’ (AOTF), a new Platform feature—developed with a user-centred vision—that enables the user to formulate more flexible therapeutic hypotheses through dynamic adjustment of the weight of contributing evidence from each source, altering the prioritisation of targets.

**Availability and implementation:**

The codebases that power the Platform—including our pipelines, GraphQL API, and React UI—are all open source and licensed under the APACHE LICENSE, VERSION 2.0. You can find all of our code repositories on GitHub at https://github.com/opentargets and on Zenodo at https://zenodo.org/records/14392214. This tool was implemented using React v18 and its code is accessible here: (https://github.com/opentargets/ot-ui-apps). The tools are accessible through the Open Targets Platform web interface (https://platform.opentargets.org/) and GraphQL API (https://platform-docs.opentargets.org/data-access/graphql-api). Data is available for download here: (https://platform.opentargets.org/downloads) and from the EMBL-EBI FTP: (https://ftp.ebi.ac.uk/pub/databases/opentargets/platform/).

## 1 Introduction

Open Targets (https://www.opentargets.org/) is a pre-competitive partnership combining expertise from academia and the pharmaceutical industry, aiming to systematically identify and prioritise targets, ultimately leading to safer, more effective therapies. One axis of the consortium’s research programme is to provide open-source data and informatics resources for the global scientific community, with the Open Targets Platform as its flagship tool (https://platform.opentargets.org/) (Buniello *et al.* 2025). The Platform harmonises, integrates, and scores publicly available evidence of target–disease associations, mapping to standardised ontologies and identifiers to enable data connectivity and verify the provenance of the data source. It provides a comprehensive knowledgebase and tooling to identify and prioritise targets, diseases, and drugs in the context of drug discovery, as well as annotations of targets, diseases, and drugs, including disease causality, target tractability and safety.

The Platform is released four times a year, ensuring regular data updates and integration of user feedback and new features. As per our latest release (24.12), the Platform includes evidence for 63 121 genes, 28 327 diseases and phenotypes, 18 041 drugs and compounds and 8 155 988 target–disease associations–for a total of >17 800 000 underlying evidence pieces that users can navigate to build their therapeutic hypotheses.

In the last few years, the Platform has undergone a complete rebuild, aiming to streamline data integration and harmonisation, enhance the visibility of the data and improve the user experience. In this context, AOTF can be seen as one of the main enhancements to the Platform user interface.

## 2 Materials and methods

Our primary goal in creating AOTF was to build a tool that could represent multiple levels of analysis on the same screen. Displaying all data source scores and evidence for individual data sources in a single screen allows the user to compare and navigate data without switching context.

### 2.1 Methodological approach and user-centred design

Our web application development process followed the Agile methodology (Meso and Jain 2006), specifically using the Scrum framework (Cervone 2011). This iterative and incremental approach enables flexibility, continuous improvement, and close collaboration among team members throughout the development lifecycle. This framework allowed the project team to gather feedback, prioritize new features or adjustments, and iteratively improve the application based on user needs and stories.

The process also followed a User-Centred Design (UCD) (Solano-Roman *et al.* 2019), emphasising the design and development lifecycle on understanding the academic and pharmaceutical communities' needs, preferences, and behaviours. Based on research and UX interviews with target users, we created detailed user personas. These personas represented diverse user segments, as well as their goals, motivations, and user flows. We then developed journey maps and low-fidelity wireframes to visualise and understand the typical paths users take when interacting with the web application.

This allowed us to iterate quickly and gather early input from stakeholders and users. Based on feedback and iterative design sessions, we ultimately created high-fidelity interactive prototypes using Figma (https://www.figma.com/prototyping/). These prototypes simulate user interface interactions, helping stakeholders and users visualise the final product.

### 2.2 Data processing and access

The AOTF tool integrates data from the Open Targets Platform through a series of structured API queries to the Open Targets GraphQL API (https://api.platform.dev.opentargets.xyz/api/v4/graphql). This API enables tailored data retrieval by allowing specific queries to request the information needed to populate AOTF widgets and views. Each component within the interface, such as disease-target association tables and evidence summaries, relies on targeted GraphQL queries that dynamically pull data as users interact with the tool. GraphQL (https://graphql.org), with its flexible query structure, enables precise access to association and evidence data, streamlining the data retrieval process. This API-driven model allows for dynamic data requests, directly facilitating real-time insight generation.

To support complex data interactions, such as dynamically assembling the AOTF view, specific GraphQL queries like associatedTargets (for disease-based queries) and associatedDiseases (for target-based queries) were used. These queries enable granular data retrieval tailored to the user’s query while reducing the overhead associated with traditional batch-processing workflows. By leveraging GraphQL's inherent schema, the API ensures that only the required data is retrieved, which is particularly advantageous in high-volume, association-driven biomedical databases like the Open Targets Platform.

Additionally, the GraphQL API allows various filtering options, including the weight parameter, to adjust the ordering of returned associations based on user input. This feature enhances the tool's interactivity, enabling users to refine their data exploration in real-time. The ability to modify the order of associations dynamically reflects a user-centred design, where various filtering options can influence the display and prioritisation of data in the resulting visualisations (Krol 2019).

### 2.3 Tool development and integration into the platform

The development and integration of this feature within the Open Targets Platform required careful selection and orchestration of multiple open-source technologies, each addressing distinct levels of application complexity. The backbone of the data layer is the Open Targets API, which leverages a GraphQL interface via Apollo GraphQL (https://www.apollographql.com/), which enables efficient querying and real-time data management.

For front-end visualisation, ReactJS (https://react.dev/) and TanStack Table (https://tanstack.com/table/latest) were strategically selected to address the need for both flexibility and performance. TanStack Table, known for its extensibility, provided a robust foundation for constructing a highly customisable table interface while managing states, transitions, and asynchronous operations within the UI. This choice facilitated the seamless incorporation of advanced filtering and sorting mechanisms, allowing users to explore associations with ease. The component-based architecture of ReactJS was instrumental in efficiently rendering the complex data outputs from TanStack Table and integrating pre-existing evidence widgets from other parts of the Platform. This reuse of components not only accelerated development but also ensured design consistency across the Platform.

On the deployment side, our approach aligned with the existing Open Targets infrastructure, utilising an NGINX (https://nginx.org/) web server hosted on Google Cloud Platform. By following the established code delivery pipeline, we streamlined the tool’s deployment process, ensuring compatibility with the broader Platform architecture while minimising operational overhead. This reuse of established deployment paths was a strategic decision to maintain coherence within the Platform’s infrastructure and leverage the scalability of Google Cloud’s services (https://cloud.google.com/).

Integration into the current Open Targets Platform, particularly within the target–disease associations page, was enabled by refactoring existing tools using ReactJS and Turbo (https://turbo.build/). This refactor allowed us to modularise the evidence widgets into reusable sections, further exposing them to the tool via a section package for AOTF. To optimise web performance and improve user experience, we used lazy loading (https://developer.mozilla.org/en-US/docs/Web/Performance/Lazy_loading) strategies, ensuring that components were loaded only when necessary. This technique minimises the initial load time, enhancing performance, especially when handling large-scale data visualisations and user interactions.

In summary, the AOTF tool development involved a considered combination of modern web development techniques and scalable infrastructure choices, resulting in a responsive, efficient, and highly integrated component of the Open Targets Platform. Each technology was selected with a specific strategy to balance performance, usability, and maintainability, ensuring that the tool can scale and evolve in parallel with the Platform’s future requirements.

### 2.4 Tool limitations

The AOTF tool has some limitations, such as the association between the data schema and the application: there is no connection between the AOTF JSON configuration and the data generated by the Open Targets Platform. This happens because the codebase hosts an object that is ‘hardcoded’ with the desired data sources that will be displayed as columns in the user interface.

Additionally, the feature is not accessible outside of a browser or without an internet connection, and—due to a conscious decision to facilitate the development process—the tool is currently only suitable for desktop-size screens. Furthermore, since the maximum length of a URL varies across different browsers, we could not use the URL for state management; however, we plan to address this in future iterations.

## 3 Results

The Associations page is the core of the Open Targets Platform web interface, providing a comprehensive view of the evidence for a given target–disease association, ranked by overall association score.

**Figure 1.**
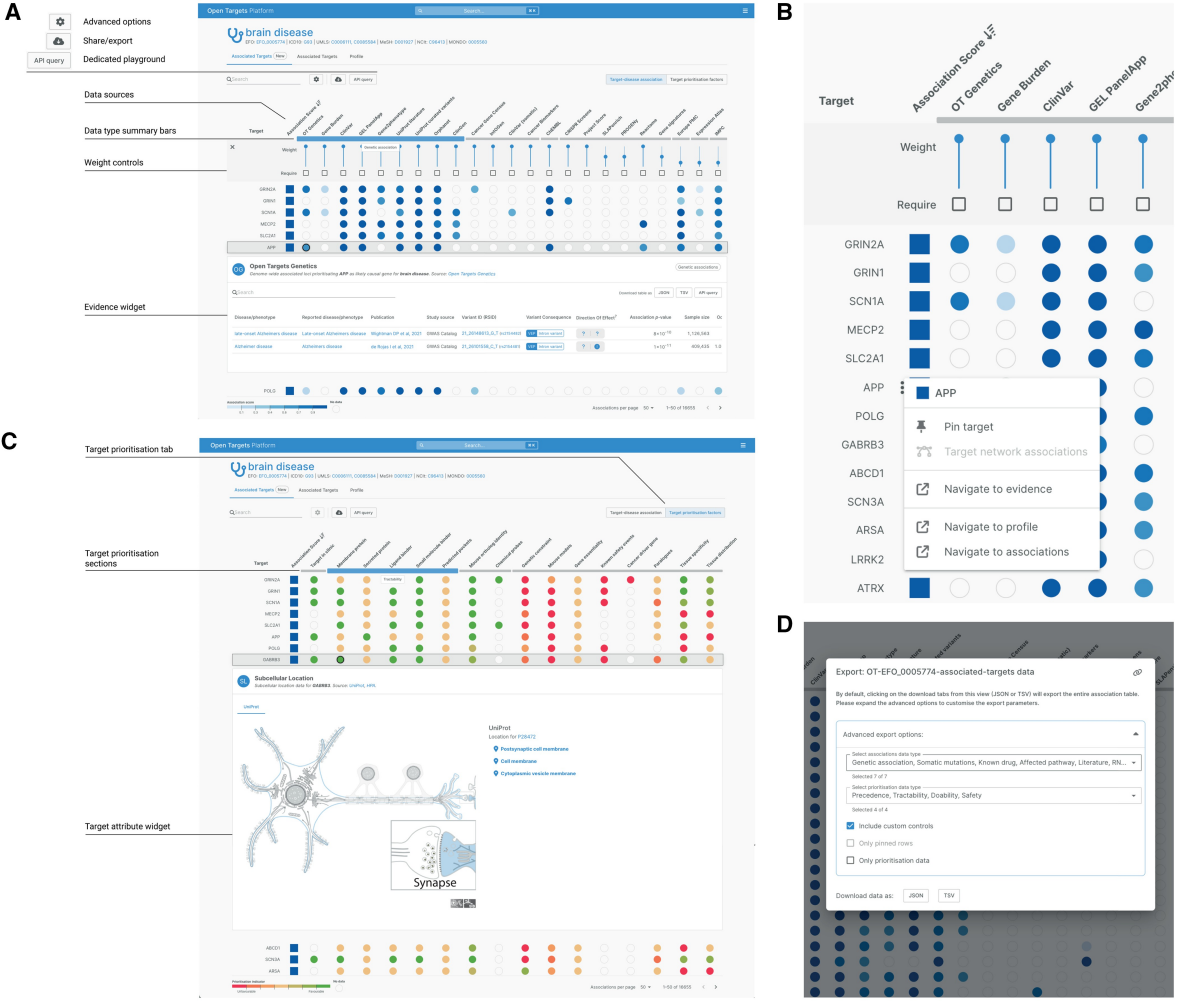
Summary panel of Associations on the Fly user interface and its subfeatures. (A) Example Association on the Fly view for brain disease (https://platform.opentargets.org/disease/EFO_0005774/associations), showing all targets associated with the disease, sorted by decreasing association score. The main features are labelled. (B) Context menu, facilitating navigation through the different Platform pages for that specific target plus target pinning (APP example). (C) Example target prioritisation view for target attributes (https://platform.opentargets.org/disease/EFO_0005774/associations?table=prioritisations), distributed from most favourable to least favourable (with arrows annotation). As highlighted in the text, a target located in the cell membrane or plasma membrane will be favourable in this view as it is likely to be more accessible to a drug(KCNB1 example). (D) Export functionality, allowing users to download the entire dataset visualized in Associations on the Fly (default status) as well as customised dataset (advanced custom controls). Since submission, the colours of the Open Targets Platform user interface have been adjusted slightly to improve accessibility.

AOTF is a revamp of this page with new, more interactive facets and additional built-in functionalities to empower users with additional control over their experience using the Platform (Fig. 1A). The feature includes a number of new applications: (i) Intuitive identification of which data sources have evidence to support a given disease/target association. (ii) Rapid comparison of evidence for different associations. (iii) Ability for users to easily modify the weighting (or relative importance) of contributing evidence from each data source. (iv) Possibility to filter by data type and data source.

AOTF was scoped, designed, and implemented with a user-centred vision. Through building user stories and UX interviews, the Open Targets community has had a large influence on the staged implementation and delivery of this product. For example, users requested the ability to prioritise target–disease associations using only genetic evidence. Through the advanced options panel in AOTF, users can now downweigh nongenetic evidence sources using the sliders or require associations to include genetic evidence by clicking on the corresponding ‘require’ boxes (Fig. 1A).

### 3.1 Association on the fly user journey

The new Associations on the Fly view is available through the Open Targets Platform by searching for a target or a disease, such as Brain Disease (Fig. 1A).

The previous version of the associations page ranked diseases or targets by their overall association score, which was then broken down into seven data type score columns (Genetic association, Somatic mutations, Known drug, Affected pathway, Literature, RNA expression, Animal model) (Koscielny *et al.* 2017). Each data type was made up of several different individual data sources. In AOTF, each column represents a single data source, giving a much more comprehensive and transparent view of the available evidence for this target–disease association. To help users navigate the data sources, the data type groupings can be viewed by hovering over the bar under the data source headers. Moreover, a pop-up book icon—appearing upon hovering over the data source headers—directs users to our comprehensive documentation describing the source.

Another innovation of AOTF is that users can browse evidence data widgets directly in the view by clicking on the buttons, supporting quick comparison of the supporting datasets, as well as the ability to easily review the provenance and strength of the underlying evidence (e.g. evidence from Open Targets Genetics in Fig. 1A).

One of the aims of this redesign—and the origin of its name—was to enable a more tailored therapeutic hypothesis formulation. Users now have the ability to modify the relative importance of the different data sources from the defaults set by Open Targets, to change the priority order of targets ‘on the fly’. This functionality can be accessed through the ‘advanced options’ tab (Fig. 1A), while additional fine-tuning is possible through an API playground. The view automatically recomputes the association scores based on the new user-defined weights, initiated by modification of the relevant API data sources ‘weight’ variable on the back-end. Filtering by data type (OR filter) is now also enabled by clicking on the data type summary bars or ‘required’ tick boxes (Fig. 1A). A context menu, accessible from each entity row when a user hovers next to the listed target or disease, has been built to allow pinning of users' favourite targets and diseases while providing easy access to profiles, associations, and evidence pages (Fig. 1B).

## 4 Conclusion

Associations on the Fly and its subview, the Target Prioritisation factor view, have been conceived with a very modular core, which enables relatively simple implementation of new developments and improvements.

For example, we have developed the beta version of an upload functionality, providing the option to upload a custom list of targets or diseases onto the two new association views. Due to the complexity and heterogeneous nature of gene and disease names, we are designing an application that will be able to suggest potential matches between the entities in the list and the ones already indexed in the Platform (Platform entity ids). When this happens, a ‘Pin hits’ tab will prompt the Associations on the Fly page to build up a custom view with the entities from the custom list (https://platform-docs.opentargets.org/web-interface/associations-on-the-fly#upload-functionality). We are also re-designing a functionality which allows filtering on the Associations On the Fly and Target Prioritisation pages.

Furthermore, following up from community requests, we plan to enable access to target interactions information within the main ‘Associations on the Fly’ view. The interactors subview will be prompted by one of the context menu options and will contain target molecular interactions from the current Platform data feeds (String, Signor, and Reactome), sorted by their individual target–disease associations score.

These features are key to enable more complex therapeutics hypotheses building, while enhancing the Open Targets Platform user experience.

## Data Availability

Features walkthrough video: https://youtu.be/2A9bksboAag and https://www.youtube.com/watch?v=WQwQn6I4jkw. Extensive documentation: https://platform-docs.opentargets.org/web-interface/associations-on-the-fly and https://platform-docs.opentargets.org/web-interface/target-prioritisation.
